# The Target MicroRNAs and Potential Underlying Mechanisms of Yiqi-Bushen-Tiaozhi Recipe against‐Non-Alcoholic Steatohepatitis

**DOI:** 10.3389/fphar.2020.529553

**Published:** 2020-11-12

**Authors:** Wei Hong, Songsong Li, Yueqin Cai, Tingting Zhang, Qingrou Yang, Beihui He, Jianshun Yu, Zhiyun Chen

**Affiliations:** ^1^ The Second Central Laboratory, The First Affliated Hospital of Zhejiang Chinese Medical University, Hangzhou, China; ^2^ Key Laboratory of Integrative Chinese and Western Medicine for the Diagnosis and Treatment of Circulatory Diseases of Zhejiang Province, Hangzhou, China; ^3^ Laboratory Animal Research Center of Zhejiang Chinese Medical University, Hangzhou, China

**Keywords:** Yiqi-Bushen-Tiaozhi recipe, Traditional Chinese Medicine, non-alcoholic fatty liver disease, non-alcoholic steatohepatitis, microRNA, ingenuity pathway analysis, network pharmacology

## Abstract

MicroRNAs (miRNAs) have emerged as potential therapeutic targets for non-alcoholic fatty liver disease/non-alcoholic steatohepatitis (NAFLD/NASH). Traditional Chineses Medicine (TCM) plays an important role in the prevention or treatment of NAFLD/NASH. However, miRNA targets of TCM against NASH still remain largely unknown. Here, we showed that Yiqi-Bushen-Tiaozhi (YBT) recipe effectively attenuated diet-induced NASH in C57BL/6 mice. To identify the miRNA targets of YBT and understand the potential underlying mechanisms, we performed network pharmacology using miRNA and mRNA deep sequencing data combined with Ingenuity Pathway Analysis (IPA). Mmu-let-7a-5p, mmu-let-7b-5p, mmu-let-7g-3p and mmu-miR-106b-3p were screened as the main targets of YBT. Our results suggested that YBT might alleviate NASH by regulating the expression of these miRNAs that potentially modulate inflammation/immunity and oxidative stress. This study provides useful information for guiding future studies on the mechanism of YBT against NASH by regulating miRNAs.

## Introduction

Non-alcoholic fatty liver disease (NAFLD) is the most common chronic liver disease worldwide and the prevalence rate is up to 20–30% (Al-Dayyat et al., 2018). In the absence of intervention, NAFLD may progress from non-alcoholic fatty liver to non-alcoholic steatohepatitis (NASH), which includes liver fibrosis, cirrhosis and hepatocellular carcinoma (HCC) (Konerman et al., 2018). Nowadays, NASH is considered as the leading cause of the end-stage liver failure (Kazankov et al., 2019). It is found that the pathogenesis of NASH is associated with the imbalance of hepatocyte lipid metabolism, oxidative stress and inflammatory response (Sanyal, 2019). However, the mechanisms involved in the progression of NASH remain to be elucidated.

MicroRNAs (miRNAs) are a class of endogenous, single-stranded non-coding RNAs with the length of ∼22-nt. miRNAs negatively regulate the expression of target mRNAs by binding to the seed region of the 3'-untranslated region of the target sequences (Agarwal et al., 2015). As a vital post-transcriptional regulation mechanism, it is one of the important ways to achieve multi-gene and multi-path regulation. Numerous miRNAs have been found to be involved in the pathogenesis of NAFLD (Iravani et al., 2018). For example, miR122 is a major regulator of hepatocyte lipid metabolism and plays a key role in the progression of hepatic steatosis to more severe liver damage such as fibrosis (Hsu et al., 2012). miR-34a is involved in many cellular physiological processes such as lipid metabolism, apoptosis, cell differentiation and cell cycle regulation (Yang et al., 2015), and plays a key role in the development of NASH (Ding et al., 2015). The functions of miR-21, miR33, and miR-125b in the pathogenesis of NAFLD were also established (Gjorgjieva et al., 2019). Therefore, miRNAs are considered as promising targets in the diagnosis and treatment of NAFLD (Dongiovanni et al., 2018; Yu et al., 2019). The miR-34a/Sirt1/p66shc pathway is currently known as the main pathway of *Rosmarinus officinalis L.* extract (Carnosic acid) against NAFLD in rats (Shan et al., 2015). The decreased expression of miR-107-3p is involved in the process of resveratrol alleviating steatosis in rats (Gracia et al., 2017). miR-103, miR-107 and miR-122 are involved in anti-NAFLD effects of the *Hibiscus sabdariffa* polyphenol extract in mice (Joven et al., 2012).

The critical functions of Traditional Chinese Medicine (TCM) have been widely reported in the prevention or treatment of NAFLD (Yao et al., 2016). However, there has been limited research on identification of miRNA therapeutic targets of TCM against NAFLD. Yiqi-Bushen-Tiaozhi (YBT) recipe is composed of Huangqi, Yingyanghuo, Fuling, Baizhu, Zhiheshouwu, Shanzha, Haizao, Yujin, Taoren. We found that YBT recipe has the effects of lowering lipid accumulation, restraining inflammation and alleviating oxidative stress, while its function in the regulation of miRNAs during NALFD remains still unclear. In this study, C57BL/6 mice were fed with Western diet (high fat and high sugar) for 16-weeks to induce NASH and treated with YBT recipe. High-throughput sequencing was performed to get a whole picture of the transcriptome and miRNA information of NASH group, YBT treated group and the control group. After the Ingenuity Pathway Analysis (IPA), the network pharmacology was used to predict the miRNA targets of YBT.

Network pharmacology plays an increasingly important role in the therapeutic mechanism and target prediction of TCM (Hong et al., 2019). IPA is an online integrated analysis software, where millions of pathway information from academic publications and public databases were mined by manual quality control. The analysis and search tools of IPA can discover the importance of the data and identify new targets or candidate biomarkers in the context of biological systems (Chowdhury and Sarkar, 2015). In the present study, to predict the miRNA targets of YBT against NASH, we performed network pharmacology by using miRNA and mRNA deep sequencing data combined with IPA. Mmu-let-7a-5p, mmu-let-7b-5p, mmu-let-7g-3p and mmu-miR-106b-3p were screened as the main targets of YBT. They may alleviate NASH symptoms by regulating inflammation/immunity and oxidative stress. Inflammation/immunity and oxidative stress are considered vital parts in the pathogenesis of NASH. Our finding suggested that mmu-let-7a-5p, mmu-let-7b-5p, mmu-let-7g-3p and mmu-miR-106b-3p may be important players in the development of NASH and might be one of the main mechanisms of YBT against NASH.

## Materials and Methods

### Preparation of the Extract Solution of Yiqi-Bushen-Tiaozhi Recipe

Each YBT recipe contains 133 g raw medicines: Huangqi 30 g, Yingyanghuo 12 g, Fuling 15 g, Baizhu 12 g, Zhiheshouwu 10 g, Shanzha 24 g, Haizao 10 g, Yujin 10 g, Taoren 10 g. The raw medicine concentration of the liquid extract for this study is 2.212 g/ml. The herbal medicines were purchase from Hangzhou Huadong Chinese Herbs Pieces Co.Ltd. The related information of the medicines (batch number, producing area and Latin name) is as follows: Huangqi: 160816, Inner Mongolia, *Astragalus membranaceus* (*Fisch.*) *Bge.var.mongholicus* (*Bge.*) *Hsiao*; Yingyanghuo: 160816, Shaanxi, *Epimedium brevicomu Maxim.*; Fuling: 161016, Zhejiang, *Poria cocos* (*Schw.*) *Wolf*; Baizhu: 160715, Zhejiang, *Atractylodes macrocephala Koidz.*; Zhiheshouwu: 160714, Zhejiang, *Polygonum multiflorum Thunb.*; Shanzha: 161013, Shandong, *Crataegus pinnatifida Bge.*; Haizao: 160829, Shandong, *Sargassum pallidum* (*Turn.*) *C.Ag.*; Yujin: 160715, Sichuan, *Curcuma phaeocaulis Val.*; Taoren: 160830, Shandong, *Prunus davidiana* (*Carr.*) *Franch.* The Latin names were searched on Chinese Pharmacopoeia (http://db.ouryao.com/yd2015/), Medicinal Plant Names Services (MPNS, https://www.kew.org/science/our-science/science-services/medicinal-plant-names-services) and the database of The Plant List (http://www.theplantlist.org/).

The extract solution of YBT recipe was prepared in the Pharmaceutial Preparation Center of the First Afﬁliated Hospital of Zhejiang Chinese Medical University. The above nine ingredients of YBT recipe were mixed in the ratio of 10:4:5:4:3:8:3:3:3, soaked with ten folds volume of water for 30 min. Boilded for 1.5 h and filtered. Added eight folds volume of water to the filter residue and boiled for 1 h. After filtration, took the filter residue, added six folds volume of water and boiled for half an hour. Combined the filtrate of the three times and concentrated to a final concentration of 2.212 crude drug gram per milliliter.

### UHPLC-MS/MS Analysis

The Ultra high performance liquid chromatography‐tandem mass spectrometry analysis was performed by Qingdao Sci-tech Innovation Quality Testing Co., Ltd. high-performance liquid chromatography (HPLC) analysis was performed on Thermo Vanquish UHPLC (Thermo Fisher Scientific). Samples of YBT extract solution was diluted with 70% methanol (1:100) was separated on an Agilent Zorbax Eclipse C18 column (1.8 μm, 2.1 × 100 mm, United States) at 4°C. The mobile phases consisted of solvents A (pure water containing 0.1% formic acid) and B (pure acetonitrile). The linear gradient elution program was performed as follows: 0.0–2.0 min, 5%B; 2.0–6.0 min, 5–30%B; 6.0–7.0 min, 30%B; 7.0–12.0 min, 30–78%B; 12.0–14.0 min, 78%B; 14.0–17.0 min, 78–95%B; 20.0–21.0 min, 95–5%B, 21.0–25.0 min, 5%B. The solvent flow rate was 300 μl/min, the column temperature was 30°C and the injection volume was 2 μl.

The MS monitoring was run on LC- MS (Q-Exactive HF, Thermo Fisher Scientific) in positive ion mode or in negative ion mode. For positive ion mode: heater temperature, 325°C; sheath gas flow rate, 45 arb; aux gas flow rate, 15 arb; sweep gas flow rate, one arb; spray voltage, 3.5 kV; capillary temperature, 330°C; S-Lens RF Level, 55%. For negative ion mode: heater temperature, 325°C; sheath gas flow rate, 45 arb; aux gas flow rate, 15 arb; sweep gas flow rate, one arb; spray voltage, 3.5 kV; capillary temperature, 330°C; S-Lens RF Level, 55%. The MS scan mode was set as Full Scan (m/z 100–1,500) and MS/MS scan mode set as dd-MS2 (TopN = 10). MS spectra were acquired at a resolution of 120,000 and MS/MS scans at 60,000. Collision mode: High energy collision dissociation.

Data analysis was performed with compound discoverer 2.1 (Thermo Scientific). Preliminary identification of the components of YBT extract solution was realized by searching databases including Thermo mzCloud, Thermo mzValut and local database.

### Experimental Animals

All procedures regarding animals in this study were in compliance and approved by the Ethics Committee of Zhejiang Chinese Medical University (Resolution number ZSLL-2016-139) and were conducted accord with the Guide for the Care and Use of Laboratory Animals (United States Department of Health). 24 male C57BL/6 mice, 6–8 weeks old, were purchased from Shanghai Sippr-BK Laboratory Animal Co. Ltd. [production license: SCXK (Hu) 2013–0016].

After 1 week of adaptive feeding, the mice were randomly divided into three groups, namely normal feeding diet (ND) group, Western diet (WD) feeding group and YBT treatment (YBT) group. Eight mice were included in each group. The mice in ND group were fed with normal feed (21.5% protein, 12.3% fat, and 66.2% carbohydrate) and had water freely. The mice in the WD group and YBT group were fed with high-fat diet (10% lard oil, 5% egg yolk powder, 2% cholesterol, 0.5% bile salt and 82.5% basic feed, W/W; 21.6% protein, 36.1% fat, 42.3% carbohydrate) and freely drank 20% fructose water for 16 weeks. From the 5th week, the mice in the YBT group were given 22.12 g/kg.d of YBT liquid extract by gavage and mice in the other groups were given an equal volume of physiological saline by gavage for 12 weeks. The feedstuff was purchased from Trophica Animal Feed High-Tech Co. Ltd. China.

All the mice of ND, WD and YBT groups were sacrificed at the end of the 16th week. The mice were fasted for 12 h before experiments (water was not restricted). Blood was taken under anesthesia. The liver specimens were collected and partially fixed in 10% neutral formalin for pathological observation and the remaining liver tissues were stored at −80°C for further analysis.

### Liver Histological Examination and Serum Parameters Measurement

Oil-red O staining and H&E staining were performed as previously described (Wu et al., 2017). NAFLD activity score (NAS) were evaluated according to the NAFLD activity scoring system (Kleiner et al., 2005). The levels of serum alanine aminotransferase, aspartate aminotransferase, total triglycerides and cholesterol were measured using commercially available kits (Diasys Diagnostic System GmbH) according to the manufacturer's standard protocols.

### High-Throughput RNA Sequencing

High-throughput RNA sequencing was performed by LC-BIO Technologies (Hangzhou) Co., Ltd. Briefly, total RNA was extracted using Trizol regent (Invitrogen, CA, United States). After quality control, the RNA was used to generate small RNA libraries and rRNA-depleted RNA libraries. The small RNA libraries were constructed according to the TruSeq Small RNA Sample Prep Kits (Illumina, San Diego, United States) protocol. rRNA-depleted RNA libraries were made using Epicentre Ribo-Zero Gold Kit (Illumina, San Diego, United States) according to the instruction manual. Small RNA and mRNA sequencing was performed on Hiseq2500 platform with 50PE strategy and Hiseq4000 platform with 150 strategy, respectively. The miRNA raw data were uploaded to the Gene Expression Omnibus database (https://www.ncbi.nlm.nih.gov/geo/query/acc.cgi?acc=GSE144721).

### Differentially Expressed MicroRNA or Differentially Expressed mRNA Identification and Canonical Pathway Enrichment by Ingenuity Pathway Analysis

The Differentially expressed miRNA (DEmiRNAs) among ND, WD and YBT groups were identified by *t*-test (*p* < 0.05). The Differentially Expressed mRNA (DEmRNAs) (*p* < 0.05 and ｜logFC｜ >1) among ND, WD and YBT groups or were screened using package edgeR (3.4.1). The heatmaps were generated using R package (3.4.1).

The predicted target genes of the DEmiRNAs were obtained from miRWalk 3.0 (binding *p* > 0.95). We took the intersection of the DEmiRNA target genes and DEmRNAs from WD vs. ND group or YBT vs. WD group to obtain the overlapping DEmRNAs in the two comparison pairs, respectively. These DEmRNAs were analysis by Shanghai Biotechnology Corporation using IPA (Qiagen, Version 49309495, Germany) to enrich the canonical pathways.

### MicroRNA-Pathway Networks Construction

The top 60 canonical pathways (20 pathways of z-score >0, 20 pathways of z-score <0 and 20 pathways of z-core unknown) in the pair-wise comparisons of WD vs. ND and YBT vs. WD were selected (*p* < 0.05), respectively. The correspondences of miRNA-target gene-pathway were established and the networks of miRNA-target gene-pathway were constructed using Cytoscape package (3.6.1). The quantity of DEmiRNA target genes enriched in a canonical pathway (namely, count-score) was calculated and DEmiRNA-canonical pathway pairs were generated. The pairs that count-score ≥2 were selected to establish the networks of miRNA-pathway using Cytoscape package (3.6.1).

### Stem-Loop qRT-PCR and mRNA qRT-PCR

Total RNA from the liver tissues of the 24 mice (n = 8/group) were extracted using RNAiso plus (Takara, Dalian, China; Cat # 9109) according to the instruction manual. Stem-loop reverse transcription and quantitative Real‐time PCR was performed to measure the expression levels of the miRNAs. Total RNA (500 ng) was reverse transcribed in a 10-μl reaction volume. The mixtures were incubated at 42°C for 60 min, 70°C for 15 min and then cooled on ice. Rnase inhibitor (Cat # 2313Q) and M-MLV (Cat # 2641A) were purchased from Takara Co., Ltd. (Dalian, China). dNTP (Cat # CD117–11) was purchased from Tiangen Biotech (Beijing) Co.,Ltd. 10 μl PCR reaction system contained 1 μl of cDNA, 0.4 μl of each primer (10 μM), 5 μl of SYBR Premix EX TaqII (TliRNaseH Plus, 2×), 0.2 μl ROX Reference Dye (50×) and 3.4 μl of water. PCR program: one cycle: 95°C, 3 min 30 cycles: 95°C, 5 s; 60°C, 45 s; 72°C 90 s. U6 was used as internal reference and three replicates for each sample. mRNA qRT-PCR was performed as we previously described and the β-actin transcripts were used as the internal control (Hong et al., 2019). Relative transcript levels were calculated via the 2^−△△C(t)^ method. The primers were synthesized by Sangon Biotech Shanghai Co., Ltd. and the sequences were shown in Supplementary Table S11.

### Liquid Suspension Array Analysis and Western Blot Assay

Total protein from the liver tissues of the 24 mice (n = 8/group) were extracted using protein extracting kit (KeyGen BioTECH, Cat # KGP2100). The protein concentrations were determined using a BCA protein quantitative kit (Multi-sciences Biotch Co., Ltd., China). Levels of protein IL-1β and CCL2 were measured using liquid suspension array according to the instruction of the MILLI-PLEX1MAP Mouse Cytokine/Chemokine Magnetic Bead kit (Cat #MCYTOMAG-70K, Merck Millipore, United States). The samples were run on Luminex 200 TM (Merck Millipore, United States) with xPONENT software. The Median Fluorescent Intensity data were analyzed using 5-parameter logistic or spline curve-ﬁtting method for calculating chemokines in samples.

The western blot were performed as we previously described (Hong et al., 2019). The antibodies of COL1A2 (abs131984-100 µg),COL3A1 (abs120021-100 µl) and TGF-β1 (EPR21143) were purchased from Absin Bioscience (Shanghai, China) or Abcam Trading Co., Ltd., and were diluted 1:5,000 or 1:1,000. The antibodies of GAPDH (Mab5465-100) and Horseradish peroxidase-conjugated immuno-globulin G antibodies (GAM0072, GAR0072) were purchased from MultiSciences Biotech, Co., Ltd., and were diluted 1:5,000. Blots were imaged and quantiﬁed using Odyssey Fc imaging system (LI-COR Biosciences). Statistical analysis

The data of qRT-PCR, Liquid suspension array analysis and Western blot assay were analyzed using SPSS17.0 software. One-way analysis of variance was used and results are reported as mean ± SD. least significant difference analysis (homogeneity of variance) was used on comparison among groups. *p* < 0.05 was statistically signiﬁcant.

## Results

**FIGURE 1:**
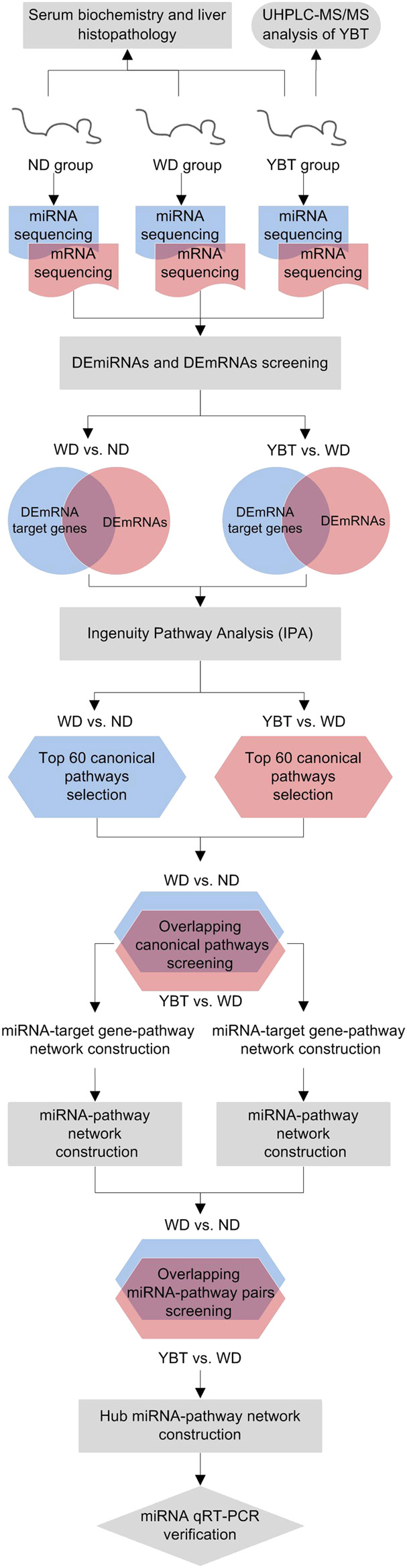
The workflow of this study. YBT, Yiqi-Bushen-Tiaozhi recipe; ND group, normal diet for 16 weeks; WD group, Western diet for 16 weeks; YBT group, Western diet for 16 weeks combined with YBT treatment for 12 weeks; UHPLC‐MS/MS, ultra high performance liquid chromatography‐tandem mass spectrometry DEmiRNAs, significantly differentially expressed miRNAs; DEmRNAs, significantly differentially expressed mRNAs.

The whole workflow of this study was summarized in Figure 1. We firstly analyzed the representative chemical compositions of YBT using HPLC. In the mean while, we performed animal experiments to assess liver injury and the effects of YBT. Next, the total hepatic RNA from mice of ND group (ND for 16 weeks), WD group (Western diet for 16 weeks) and YBT group (Western diet for 16 weeks combined with YBT intervention for 12 weeks) were extracted and subjected to high-throughput sequencing. The network pharmacology analysis was as follows: we started with analyzing the significantly DEmiRNAs and DEmRNAs in WD vs. ND group or YBT vs. WD group. Then, we took the intersection of the DEmiRNA target genes and DEmRNAs in WD vs. ND group or in YBT vs. WD group respectively to find the overlapping genes. These genes were subjected to IPA to obtain the canonical pathways, respectively. In IPA system, the activities of the canonical pathways are represented by the z-scores. According to the z-scores, we selected 60 pathways from WD vs. ND group and YBT vs. WD group for further analysis, respectively. After screening the overlapping canonical pathways between WD vs. ND and YBT vs. WD groups, the miRNA-target gene-pathway networks were generated. Then the networks were simplified into miRNA-pathway networks according to the count-score (see section below). After screening overlapping miRNA-pathway pairs between WD vs. ND and YBT vs. WD networks, hub miRNA-pathway network was generated and qRT-PCR was performed to verify the hub miRNAs.

### UHPLC-MS/MS Analysis

**FIGURE 2:**
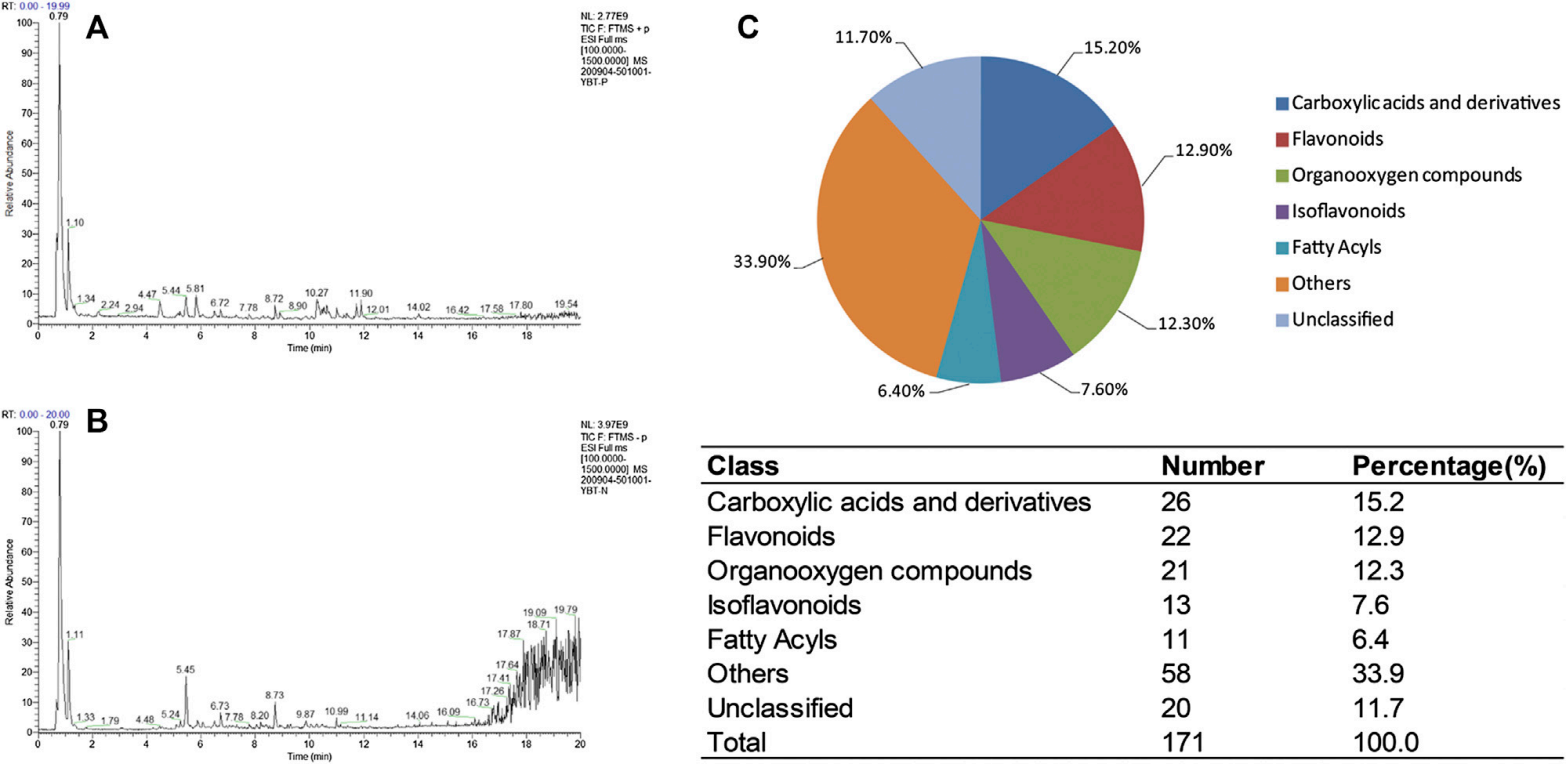
UHPLC-MS/MS of Yiqi-Bushen-Tiaozhi extract solution. **(A)**. Total ion current (TIC) chromatograms in positive mode. **(B)**. TIC chromatograms in negative mode. **(C)**. Classification of the bioactive compounds. The pie graph depicts the percentage of each classification and the number of bioactive compounds in each was shown at the bottom.

Identification of bioactive compounds in extract solution of YBT recipe were performed using UHPLC-MS/MS (Figure 2). A total of 171 substances were identified in positive ion mode and negative ion mode (Supplementary Table S1). Among them, 15.2% bioactive compounds were classified to carboxylic acids and derivatives, 12.9% to flavonoids, 12.3% to organooxygens, 7.6% to isoflavonoids, and 6.4% to fatty acyls. Furthermore, there were 33.9% classified to other classes and 11.7% were unclassified.

### Yiqi-Bushen-Tiaozhi Ameliorated Hepatic Steatosis and Inflammation in Mice Induced by Western Diet

**FIGURE 3:**
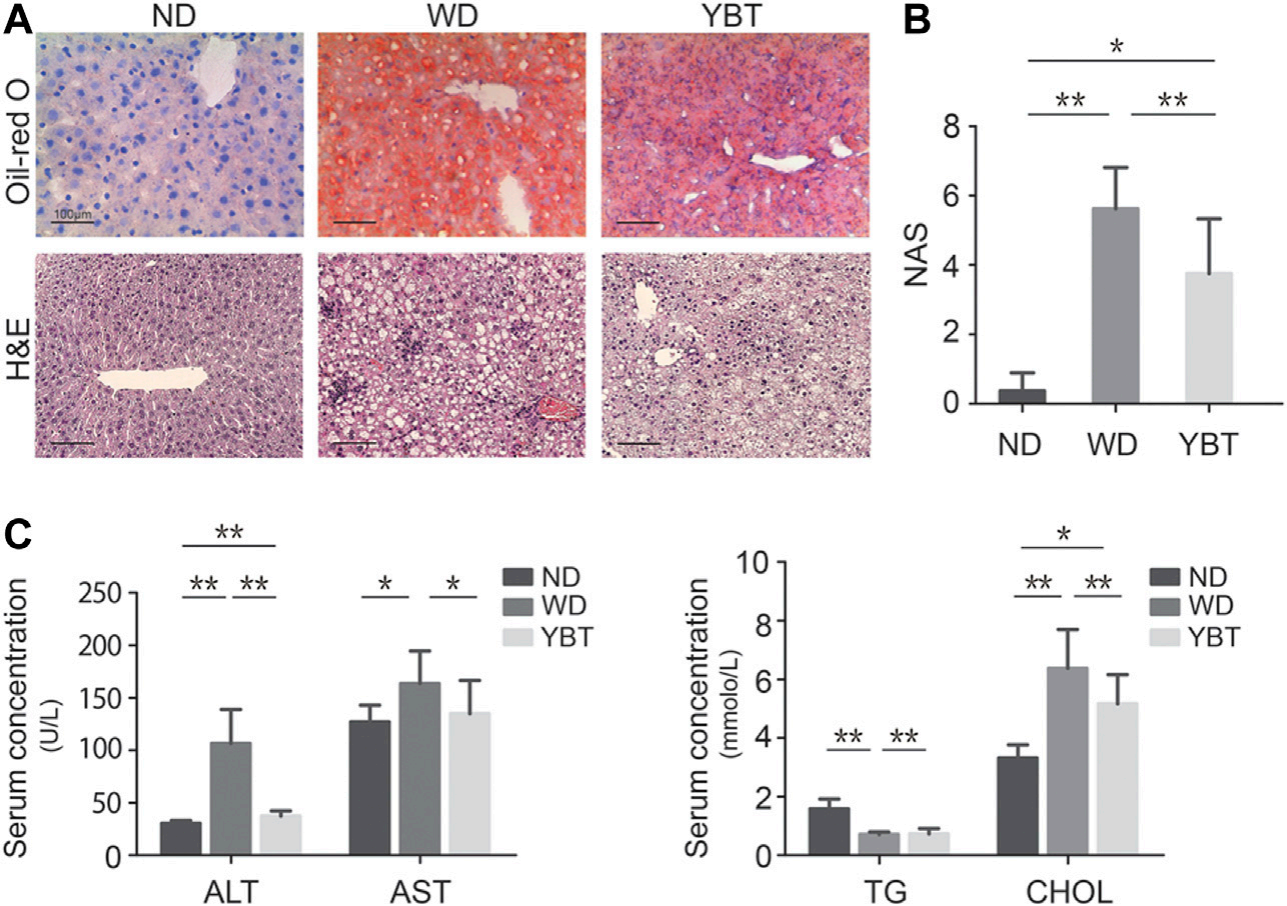
Yiqi-Bushen-Tiaozhi (YBT) ameliorated hepatic steatosis and inflammation in mice induced by WD. **(A)**. Representative Oil-red O stained **(top line)** and H&E stained **(bottom line)** liver sections (Scale bar = 100 μm). **(B)**. NAFLD activity score (NAS) evaluation of ND, WD and YBT groups. **(C)**. Serum concentrations of ALT, AST **(left)** and TG, CHOL **(right)**. ND, normal diet for 16 weeks; WD, Western diet for 16 weeks; YBT, Western diet for 16 weeks combined with YBT treatment for 12 weeks. Results are shown as mean ± SD (n = 8). *p < 0.05, **p < 0.01.

To assess the effects of YBT on hepatic steatosis and inflammation levels, mice liver sections were firstly stained by Oil-red O and H&E. The result of Oil-red O staining showed that, the levels of hepatic lipid accumulation of the WD-fed mice were much higher than that of the ND-fed mice. Compared with WD-fed mice, the levels of hepatic lipid accumulation in YBT-treated mice were much lower (Figure 3A top line). H&E staining revealed increased adipocyte size and more inflammatory cell infiltration in WD-fed mice compared with ND-fed mice. In YBT-treated mice liver section, H&E staining displayed decreased adipocyte size and a smaller quantity of inflammatory cell infiltration (Figure 3A bottom line). Then the H&E-stained liver sections were evaluated byNAS scoring system (Choi et al., 2019). NAS of WD group was much higher than that of ND group. NAS of YBT group was significantly reduced compared with WD group (Figure 3B). The serum biochemistry results showed that the levels of serum ALT, AST and CHOL were significantly increased in the WD group compared with ND group. These three parameters were significantly decreased after YBT intervention. The serum TG level of the WD group was significantly lower than that of ND group. Compared with WD group, the serum TG level was significantly increased in YBT group (Figure 3C).

### Identification of Differentially Expressed MicroRNAs or Differentially Expressed mRNAs

**FIGURE 4:**
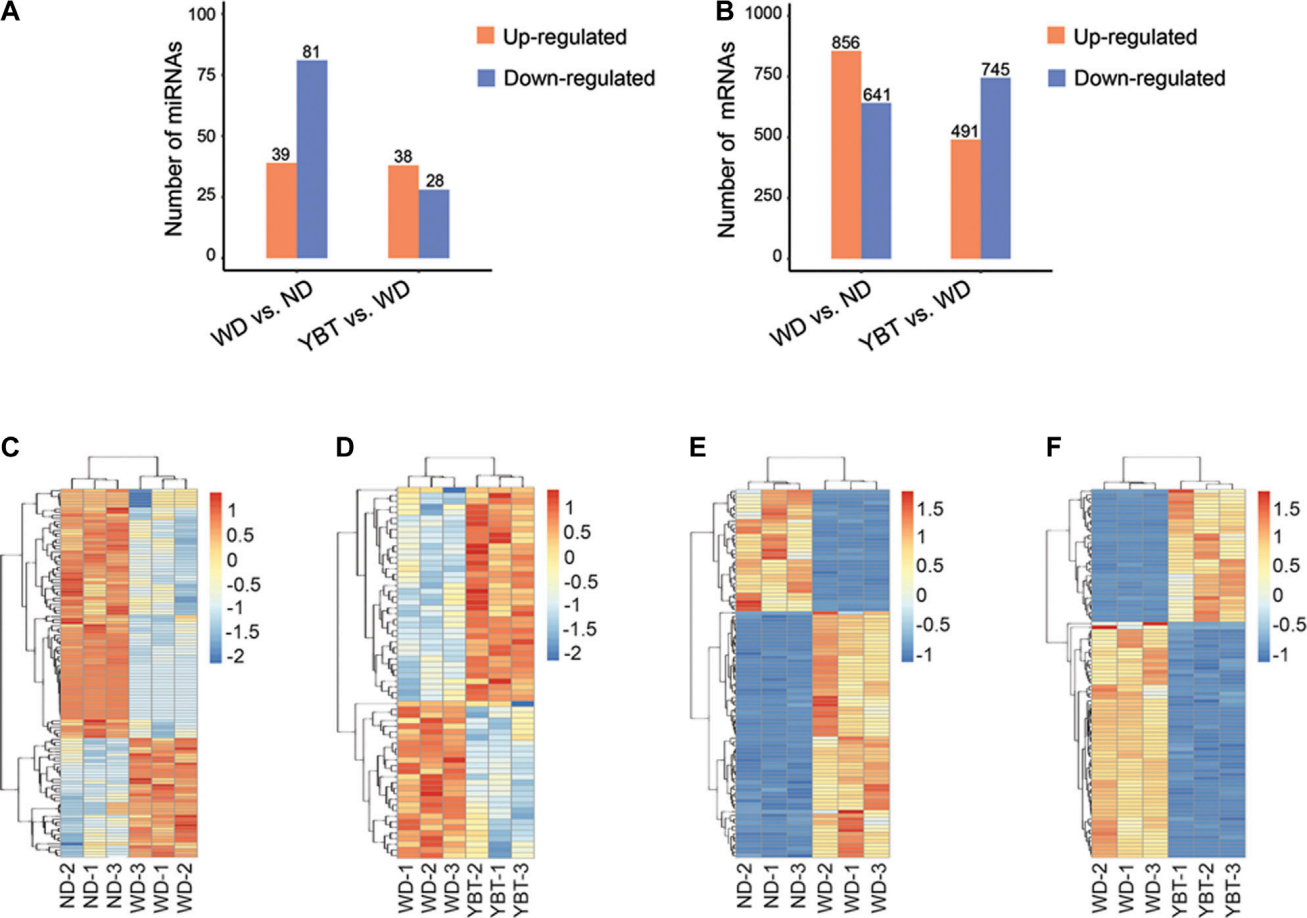
Global analysis of DEmiRNAs and DEmRNAs of WD vs. ND group or Yiqi-Bushen-Tiaozhi (YBT) vs. WD group. **(A,B)**. The bar-charts showing the numbers of up-regulated and down-regulated DEmiRNAs or DEmRNAs. **(C,D)**. The heatmaps of DEmiRNA expression profiles (*p* < 0.05). **(E,F)**. The heatmaps of DEmRNA expression profiles (*p* < 0.05, top 100). Scale bar denotes Z values. DEmiRNAs, significantly differentially expressed MicroRNAs; DEmRNAs, significantly differentially expressed mRNAs; ND, normal diet for 16-weeks; WD, Western diet for 16-weeks; YBT, Western diet for 16 weeks combined with YBT treatment for 12 weeks.

The miRNA and mRNA of the mice from the above three groups were extracted and subjected to high-through sequencing. Firstly, we identified the DEmiRNAs and DEmRNAs among ND, WD and YBT groups, and compared the expression pattern between the three groups. The clustering results showed that compared with WD group, the expression patterns of DEmiRNA and DEmRNA in ND group and YBT group were more similar (Supplementary Figure S1). Next, we analyzed the DEmiRNAs and DEmRNAs in WD vs. ND group or YBT vs. WD group. In total, 120 DEmiRNAs were identified in WD vs. ND group, among which 39 miRNAs were up-regulated and 81 miRNAs were down-regulated (Figures 4A,C; Supplementary Table S2). 66 miRNAs were significantly differentially expressed in YBT vs. WD group including 38 increased miRNAs and 28 decreased miRNAs (Figures 4A,D; Supplementary Table S2). In WD vs. ND group, there were 1,497 mRNAs significantly different expressed. Among them, the expression of 856 mRNAs were increased and 641 mRNAs were decreased (Figures 4B,E; Supplementary Table S3). One thousand two hundred thirty six DEmRNAs were identified in YBT vs. WD group, in which 491 were up-regulated and 745 were down-regulated (Figures 4B,F; Supplementary Table S3).

### Ingenuity Pathway Analysis

**FIGURE 5:**
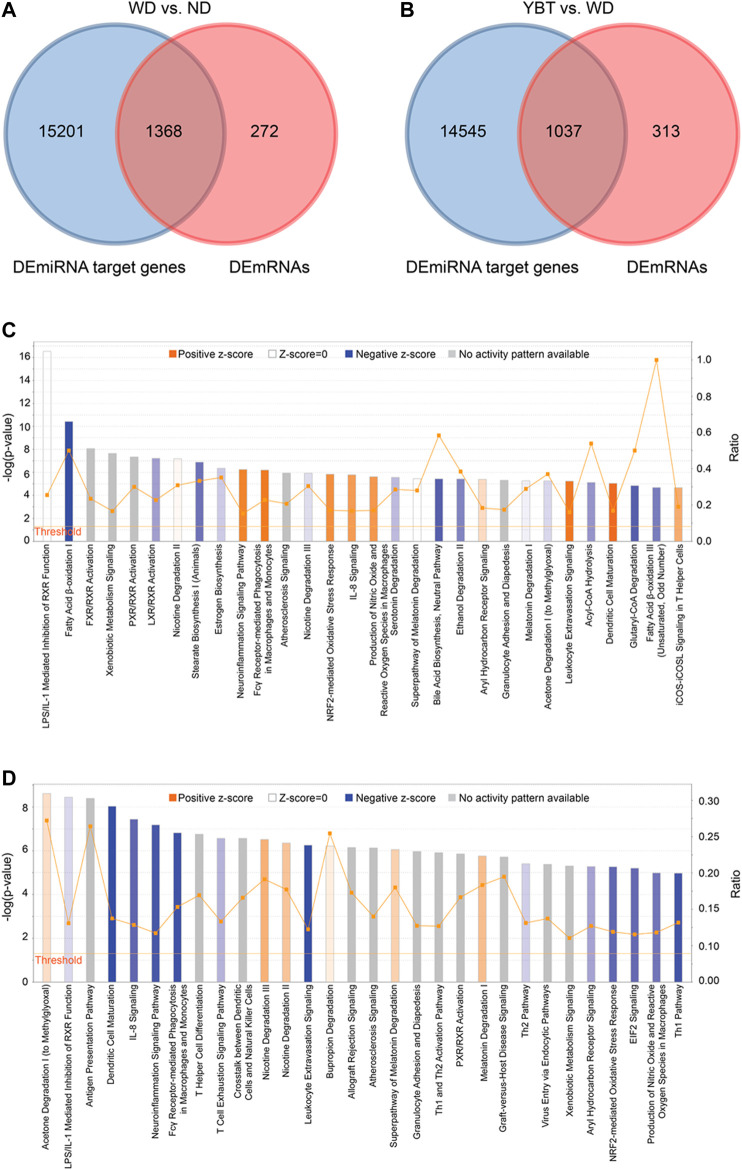
The canonical pathways enriched by Ingenuity Pathway Analysis (IPA). **(A,B)**. The Venn diagrams showing the overlapping genes between Differentially Expressed miRNA target genes and Differentially Expressed mRNA (DEmRNAs) in Western diet (WD) vs. normal diet (ND) group or Yiqi-Bushen-Tiaozhi vs. WD group. **(C)**. The canonical pathways enriched by IPA from the intersection data of panel 5A **(D)** The canonical pathways enriched by IPA from the intersection data of panel 5B. The pathways were ranked in the order of –log (*p*-value) and the top 30 of them were showed here. Threshold, when *p*-value < 0.05, –log (*p*-value) > 1.3; Ratio, the proportion of the DEmRNAs in the total genes of a pathway.

A total of 1,368 overlapping genes were obtained by taking the intersection of the DEmiRNA target genes and DEmRNAs in the WD vs. ND group (Figure 5A; Supplementary Table S4). We took the intersection of the DEmiRNA target genes and DEmRNAs in the YBT vs. WD group and obtained 1037 DEmRNAs (Figure 5B; Supplementary Table S4). Canonical pathways enrichments were conducted with the overlapping DEmRNAs from the above two intersecections using IPA, respectively (Figures 5C,D).

In IPA system, the activity of a canonical pathway is represented by the z-score. A positive z-score indicates increased functional activity of a pathway; a negative z-score means decreased functional activity of a pathway; z-score of zero or an unknown z-score denotes no activity changed in the pathway or no activity pattern is available in the current database.

Totally 175 canonical pathways (*p* < 0.05) were enriched from WD vs. ND group (Supplementary Table S5). Among them, there were 56 pathways with positive z-score, including pathways of Dendritic Cell Maturation (z-score 4.9), Leukocyte Extravasation Signaling (z-score 3.9), Fcγ Receptor-mediated Phagocytosis in Macrophages and Monocytes (z-score 3.71), etc. There were 52 pathways with negative z-score, including pathways of Fatty Acid β-oxidation I (z-score −4), Superpathway of Cholesterol Biosynthesis (z-score −3), Antioxidant Action of Vitamin C (z-score −2.828), etc. There were 10 pathways with z-score of zero and 57 pathways with z-score unknown (Table 1; Supplementary Table S5).

**TABLE 1:** z-scores of the canonical pathways.

z-score	WD vs. ND	YBT vs. WD
Positive (increased activity)	56	36
Negative (decreased activity)	52	111
Zero (no activity changed)	10	2
Unkown (no activity pattern available)	57	71
Total	175	220

ND, mice were fed with normal diet for 16-weeks; WD, mice were fed with Western diet for 16 weeks; YBT, mice were fed with Western diet for 16 weeks combined with YBT for 12 weeks.

From the comparison pair of YBT vs. WD, 220 canonical pathways were obtained (*p* < 0.05). There were 36 pathways with positive z-score and 111 pathways with negative z-score, including pathways of Fatty Acid β-oxidation I (z-score 2.646), Antioxidant Action of Vitamin C (z-score 2.646), PD-1, PD-L1 cancer immunotherapy pathway (z-score 2.5), Dendritic Cell Maturation (z-score −4.6), Leukocyte Extravasation Signaling (z-score −4.315), Fcγ Receptor-mediated Phagocytosis in Macrophages and Monocytes (z-score −3.15),etc. There were two pathways with z-score of zero and 71 pathways with z-score unknown (Table 1; Supplementary Table S5).

### Selection of the Canonical Pathways and Construction of miRNA-Target Gene Pathway Networks

We selected the following three types of canonical pathways for further analysis: positive z-score, top20 (ranked by z-score, *p* < 0.05); negative z-score, top 20 (ranked by z-score, *p* < 0.05), z-score unknown, top 20 (ranked by *p*-value, *p* < 0.05) (Table 2; Supplementary Table S6).

**TABLE 2:** The general information of the selected canonical pathways.

Comparison pair	z-score > 0	z-score < 0	z-score unknown	Total
WD vs. ND	20	20	20	60
Yiqi-Bushen-Tiaozhi vs. WD	20	20	20	60

WD, Western diet; ND, normal diet. When z-score > 0 or < 0, the top 20 pathways were ranked by z-score, *p* < 0.05; when z-score unknown, the top 20 pathways were ranked by *p*-value, *p* < 0.05.

**FIGURE 6:**
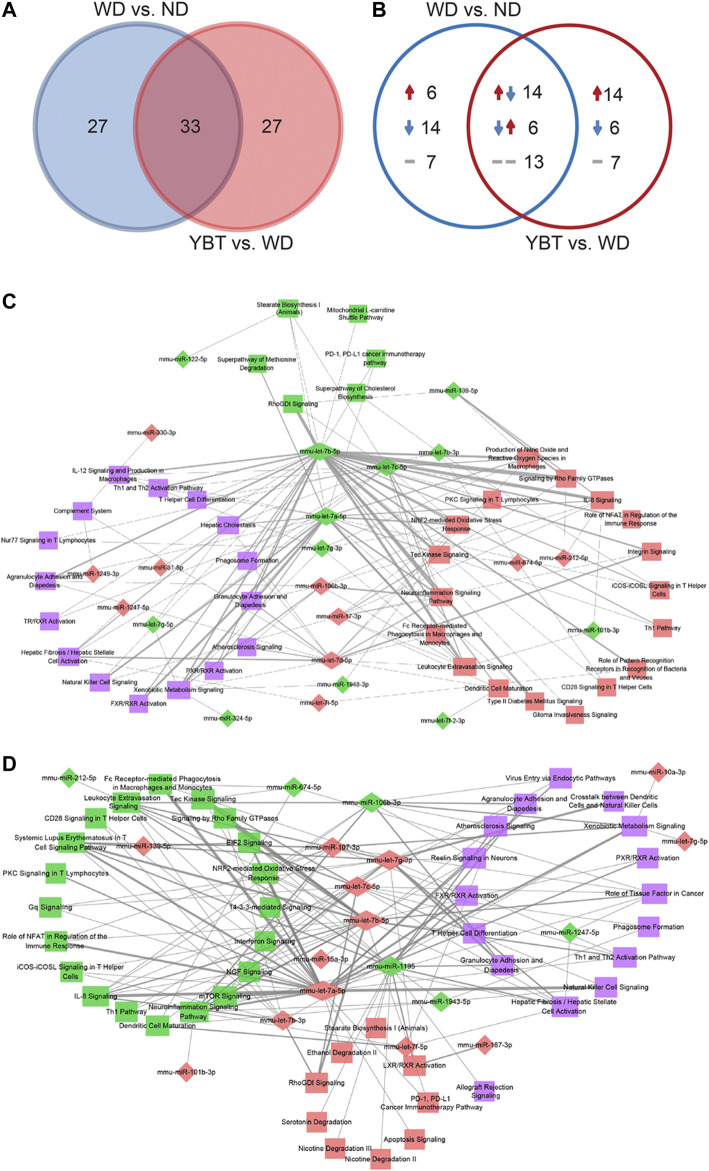
The venn diagram analysis of the overlapping canonical pathways and construction of MicroRNAs (miRNA)-pathway networks. **(A)**. The venn diagram illustrating the total number of the overlapping canonical pathways between Western diet (WD) vs. normal diet (ND) group and Yiqi-Bushen-Tiaozhi (YBT) vs. WD group. **(B)**. The venn diagram showing the activities of the canonical pathways of the two comparison pairs. The numbers indicate the count of the canonical pathways. The upward or downward arrow indicates z-scores >0 or <0, respectively. The short horizontal line denotes the z-score was unknown. **(C)**. The miRNA-pathway network of WD vs. ND group (count-score ≥2). **(D)**. The miRNA-pathway network of YBT vs. WD group (count-score ≥2). The diamond or rectangle node indicates miRNA or pathway, respectively. Red indicates up-regulation or z-score >0; green indicates down-regulation or z-score < 0; purple indicates z-score unknown. The thickness of the edge represents the count-score (the quantity of Differentially Expressed miRNA target genes enriched in a canonical pathway).

From the above two comparison pairs, we obtained 33 common canonical pathways (Figure 6A; Supplementary Table S7). Among them, 14 pathways were up-regulated in WD vs. ND group (z-score >0) while down-regulated in YBT vs. WD group (z-score <0); six pathways were down-regulated in WD vs. ND group (z-score <0) while up-regulated in YBT vs. WD group (z-score >0). There were 13 pathways that no activity pattern available in the IPA database (Figure 6B; Supplementary Table S7).

We integrated the DEmiRNA-target gene pairs and DEmRNA-canonical pathway pairs and generated miRNA-target gene-pathway networks using Cytoscape package. The network of WD vs. ND contained 468 nodes (340 target genes, 68 miRNAs, 60 canonical pathways) and 1,269 edges (Supplementary Figure S1A; Supplementary Table S8). Total 326 nodes (224 target genes, 42 miRNAs, 60 canonical pathways) and 953 edges were involved in the network of YBT vs. WD (Supplementary Figure S1B; Supplementary Table S8).

### Construction of MicroRNAs-Pathway Networks

Based on the miRNA-target gene-pathway networks, we calculated the quantity of DEmiRNA target genes enriched in a canonical pathway (namely, count-score) and generated DEmiRNA-canonical pathway pairs (Supplementary Table S9). Then we selected the pairs with count-score ≥2 to establish the networks of miRNA-pathway using Cytoscape package (Supplementary Table S10). Figure 6C shows the miRNA-pathway network of WD vs. ND. This network contained 62 nodes (22 miRNAs, 40 pathways) and 137 edges. YBT vs. WD network showed in Figure 6D. This network consisted of 64 nodes (19 miRNAs, 45 pathways) and 148 edges.

We performed a topological analysis on the networks and calculated the connectivity degrees between each DEmiRNA node and each canonical pathway node. The higher the connectivity degree of a node, the more important it is. Table 3 shows the top 10 DEmiRNAs or canonical pathways. In WD vs. ND network, the connectivity degrees of mmu-let-7b-5p, mmu-let-7a-5p and mmu-let-7c-5p were 34, 23 and 16, respectively, which suggested that mmu-let-7b-5p, mmu-let-7a-5p and mmu-let-7c-5p regulated 34, 23 and 16 pathways, respectively. The connectivity degrees of pathways of IL-8 signaling, Leukocyte extravasation signaling, NRF2-mediated oxidative stress response and Xenobiotic metabolism signaling pathways were eight, which indicated that the pathways were regulated by eight miRNAs, respectively. In YBT vs. WD network, mmu-let-7a-5p, mmu-let-7b-5p and mmu-let-7g-3p regulated 28, 21 and 17 pathways, respectively. Pathways of Neuroinflammation signaling, Leukocyte extravasation signaling and IL-8 signaling were regulated by 9, 8 and 7 miRNAs, respectively.

**TABLE 3:** Top 10 Differentially Expressed miRNAs or canonical pathways in the miRNA-pathway network of WD vs. ND or YBT vs. WD.

Network	Node (miRNA)	miRNA activity	Connectivity degree	Node (pathway)	Pathway activity	Connectivity degree
WD	mmu-let-7b-5p	↓	34	IL-8 Signaling	↑	8
vs.	mmu-let-7a-5p	↓	23	Leukocyte Extravasation Signaling	↑	8
ND	mmu-let-7c-5p	↓	16	NRF2-mediated Oxidative Stress Response	↑	8
	mmu-let-7days-5p	↑	15	Xenobiotic Metabolism Signaling	—	8
	mmu-miR-17-3p	↑	9	Neuroinflammation Signaling Pathway	↑	6
	mmu-miR-106b-3p	↑	8	Production of Nitric Oxide and Reactive Oxygen Species in Macrophages	↑	6
	mmu-let-7g-3p	↓	6	Hepatic Cholestasis	—	6
	mmu-miR-139-5p	↓	4	Tec Kinase Signaling	↑	5
	mmu-let-7b-3p	↓	4	Hepatic Fibrosis/Hepatic Stellate Cell Activation	—	5
	mmu-miR-212-5p	↑	2	Signaling by Rho Family GTPases	↑	4
YBT	mmu-let-7a-5p	↑	28	Neuroinflammation Signaling Pathway	↓	9
vs.	mmu-let-7b-5p	↑	21	Leukocyte Extravasation Signaling	↓	8
WD	mmu-let-7g-3p	↑	17	IL-8 Signaling	↓	7
	mmu-miR-1195	↓	16	Systemic Lupus Erythematosus In T Cell Signaling Pathway	↓	7
	mmu-let-7c-5p	↑	15	Hepatic Fibrosis/Hepatic Stellate Cell Activation	—	7
	mmu-miR-106b-3p	↓	10	NRF2-mediated Oxidative Stress Response	↓	6
	mmu-miR-107-3p	↑	8	Xenobiotic Metabolism Signaling	—	6
	mmu-let-7b-3p	↑	8	Role of Tissue Factor in Cancer	—	6
	mmu-miR-1943-5p	↓	5	mTOR Signaling	↓	4
	mmu-miR-674-5p	↓	4	Tec Kinase Signaling	↓	4

miRNA, MicroRNAs; WD, Western diet; ND, normal diet; YBT, Yiqi-Bushen-Tiaozhi. Connectivity degree, the number of edges between two nodes; upward or downward arrow, up-regulation or down-regulation; short horizontal line, no activity pattern available. *p* < 0.05.

### Screening of hub MicroRNAs

**FIGURE 7:**
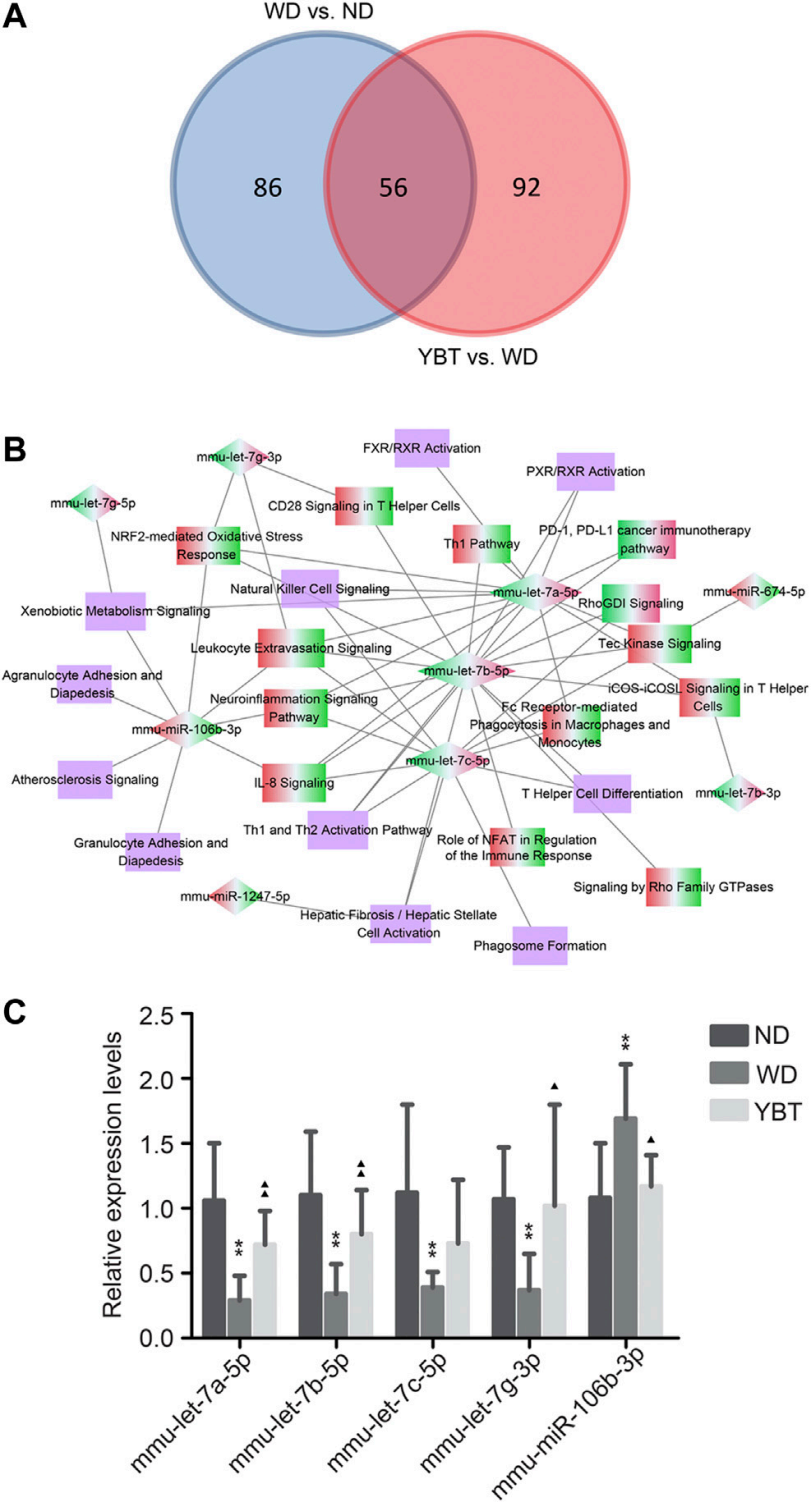
Screening the hub MicroRNAs (miRNAs) and qRT-PCR verification. **(A)**. Venn diagram depicting the overlapping miRNA-pathway pairs between Western diet (WD) vs. normal diet (ND) network and Yiqi-Bushen-Tiaozhi (YBT) vs. WD network. **(B)**. Hub miRNA-pathway network generated on the basis of intersection data of panel 7A. The diamond denotes the hub miRNA and the rectangle represents the canonical pathway. The red-green color presents the node was up-regulated in WD vs. ND group and down-regulated in YBT vs. WD group; the green-red color indicates the opposite situation. **(C)**. Stem-loop qRT-PCR verification of hub miRNAs expression levels. U6 was used as the internal control; compared with ND group, ^∗^ *p* < 0.05, ^∗∗^ *p* < 0.01; compared with WD group, ^▲^ *p* < 0.05, ^▲▲^ *p* < 0.01; n = 8/group.

To obtain the potential target miRNAs of YBT against NASH, we first screened the overlapping miRNA-pathway pairs between WD vs. ND and YBT vs. WD networks. We took the intersection of the miRNA-pathway pairs between WD vs. ND and YBT vs. WD networks and 56 miRNA-pathway pairs were obtained (Figure 7A). Next, we generated hub miRNA-pathway network including nine hub miRNAs and 24 canonical pathways (Figure 7B). Among them, mmu-let-7b-5p, mmu-let-7a-5p, mmu-let-7c-5p, mmu-miR-106b-3p and mmu-let-7g-3p were the top five miRNAs with the highest connectivity degrees (Table 4). Mmu-let-7b-5p, mmu-let-7a-5p, mmu-let-7c-5p, and mmu-let-7g-3p were down-regulated in WD vs. ND group and up-regulated in YBT vs. WD group, while mmu-miR-106b-3p was up-regulated in WD vs. ND group and down-regulated in YBT vs. WD group. Table 4 also shows that pathways of NRF2-mediated oxidative stress response, IL-8 signaling and Leukocyte extravasation signaling were of the highest connectivity degrees. They were up-regulated in WD vs. ND group and down-regulated in YBT vs. WD group. Moreover, there were 11 pathways with unknown activities.

**TABLE 4:** Nodes information of the hub miRNA-pathway network.

Node (miRNA/canonical pathway)	Connectivity degree	Activity
mmu-let-7b-5p	16	↓↑
mmu-let-7a-5p	14	↓↑
mmu-let-7c-5p	10	↓↑
mmu-miR-106b-3p	8	↑↓
mmu-let-7g-3p	3	↓↑
mmu-let-7g-5p	1	↓↑
mmu-miR-1247-5p	1	↑↓
mmu-let-7b-3p	1	↓↑
mmu-miR-674-5p	1	↑↓
NRF2-mediated Oxidative Stress Response	4	↑↓
IL-8 Signaling	4	↑↓
Leukocyte Extravasation Signaling	4	↑↓
Neuroinflammation Signaling Pathway	3	↑↓
Tec Kinase Signaling	3	↑↓
iCOS-iCOSL Signaling in T Helper Cells	3	↑↓
Th1 and Th2 Activation Pathway	3	—
Xenobiotic Metabolism Signaling	3	—
Th1 Pathway	2	↑↓
CD28 Signaling in T Helper Cells	2	↑↓
PXR/RXR Activation	2	—
PD-1, PD-L1 cancer immunotherapy pathway	2	↓↑
RhoGDI Signaling	2	↓↑
Fc Receptor-mediated Phagocytosis in Macrophages and Monocytes	2	↑↓
T Helper Cell Differentiation	2	—
Hepatic Fibrosis/Hepatic Stellate Cell Activation	2	—
Natural Killer Cell Signaling	2	—
Agranulocyte Adhesion and Diapedesis	1	—
Atherosclerosis Signaling	1	—
FXR/RXR Activation	1	—
Signaling by Rho Family GTPases	1	↑↓
Role of NFAT in Regulation of the Immune Response	1	↑↓
Phagosome Formation	1	—
Granulocyte Adhesion and Diapedesis	1	—

Connectivity degree, the number of edges between two nodes; up- or down-ward arrow, a MicroRNAs or a canonical pathway is up- or down-regulated; short horizontal line, the activity of a pathway is unkown. *p* < 0.05.

### qRT-PCR Verification

The expression levels of the hub miRNAs with connectivity degree ≥3 (Figure 7B; Table 4) were validated by stem-loop qRT-PCR. Compared with the ND group, the expression levels of mmu-let-7a-5p, mmu-let-7b-5p, mmu-let-7c-5p and mmu-let-7g-3p of the WD group were significantly down-regulated, while the expression level of mmu-miR-106b-3p was significantly up-regulated in WD group. After YBT intervention, except for mmu-let-7c-5p, the expression levels of the other four miRNAs, mmu-let-7a-5p, mmu-let-7b-5p, mmu-let-7g-3p and mmu-miR-106b-3p were significantly changed compared with the WD group (Figure 7C).

## Discussion

Network pharmacology has become an important means to predict key targets for TCM (Zhang et al., 2019). Our previous study predicted VEGF-C as a key potential target of PTFC (Pure total ﬂavonoids from Citrus) against NAFLD via network pharmacology (Hong et al., 2019). As a powerful tool for bioinformatics analysis, IPA is an important player in analyzing drug mechanism of action. In this study, we showed that YBT recipe effectively ameliorated NASH symptoms in mice (Figure 3). To identify the target miRNAs of YBT against NASH, we integrated miRNA and mRNA sequencing data using IPA and performed pharmacology network analysis. The connections between hub miRNAs and the canonical pathways were established (Figure 7B).

qRT-PCR verification (Figure 7C) showed that, the expression levels of all the five of the predicted hub miRNAs, mmu-let-7a-5p, mmu-let-7b-5p, mmu-let-7c-5p, mmu-let-7g-3p and mmu-miR-106b-3p, significantly changed in NASH mice. Compared with the WD group, the expression levels of mmu-let-7a-5p, mmu-let-7b-5p, mmu-let-7g-3p and mmu-miR-106b-3p other than mmu-let-7c-5p showed significant changing in the YBT group. The result suggested that all the five predicted hub miRNAs may participate in the development of NASH and four of them might be associated with the effects of YBT. Therefore, mmu-let-7a-5p, mmu-let-7b-5p, mmu-let-7g-3p and mmu-miR-106b-3p might be the core potential miRNA therapeutic targets of YBT against NASH. This result also indicated that the prediction in this study showed high accuracy.

Let-7a-5p, let-7b-5p and let-7g-3p belong to Let-7 family (Ustianenko et al., 2018). Let-7a-5p (namely, let-7) and let-7b-5p (namely, let-7b) exhibit similar activities in HCC and hepatic fibrosis. Expression levels of let-7a-5p (Balzeau et al., 2017) and let-7b-5p (Gerard et al., 2019) are down-regulated in HCC. The functions of let-7a-5p (Cai et al., 2018; Matsuura et al., 2018) and let-7b-5p (McDaniel et al., 2017; Tang et al., 2017) in hepatic fibrosis have been well documented. Overexpression of let-7a and let-7b suppress the myofibroblastic activation of cultured human hepatic stellate cells induced by LPS and TGF-β (McDaniel et al., 2017; Zhang et al., 2019). In our study, the levels of mmu-let-7a-5p and mmu-let-7b-5p significantly reduced in NASH mice, which is consistent with the above reports that let-7a-5p and let-7b-5p were down-regulated in HCC or hepatic fibrosis samples. This result suggested that let-7a-5p and let-7b-5p may have similar functions in NASH development. YBT intervention significantly increased their expression levels, which indicated that YBT alleviated the aberrant expression of mmu-let-7a-5p and mmu-let-7b-5p. To the best of our knowledge, the activity of let-7g-3p in liver disease has not been reported. In our study, mmu-let-7g-3p was down-regulated in NASH mice and up-regulated in YBT treated mice, which suggesting that it may participate in NASH progression and is regulated by YBT.

Interestingly, mmu-miR-106b-3p was the only one target miRNA that was up-regulated in NASH mice and down-regulated in YBT treated mice. Abnormal expression of miR-106b-3p has been found in several types of cancers (Lu et al., 2014; Wang et al., 2017), which is associated with cell cycle (Vardarli et al., 2018), cell proliferation and mesenchymal transition (Qiao et al., 2019). Recently, it was found that, miR-106b-3p was down-regulated when human retinal pigment epithelium cell was incubated with H_2_O_2_ (Ayaz and Dinc, 2018), while circulating miR-106b-3p exhibits an increased level in HCC patients compared with cirrhosis patient or healthy control (Moshiri et al., 2018). Our result demonstrated that the activity of mmu-miR-106b-3p is in agreement with circulating miR-106b-3p in HCC patients and YBT intervention suppressed the abnormal expression of mmu-miR-106b-3p.

**FIGURE 8:**
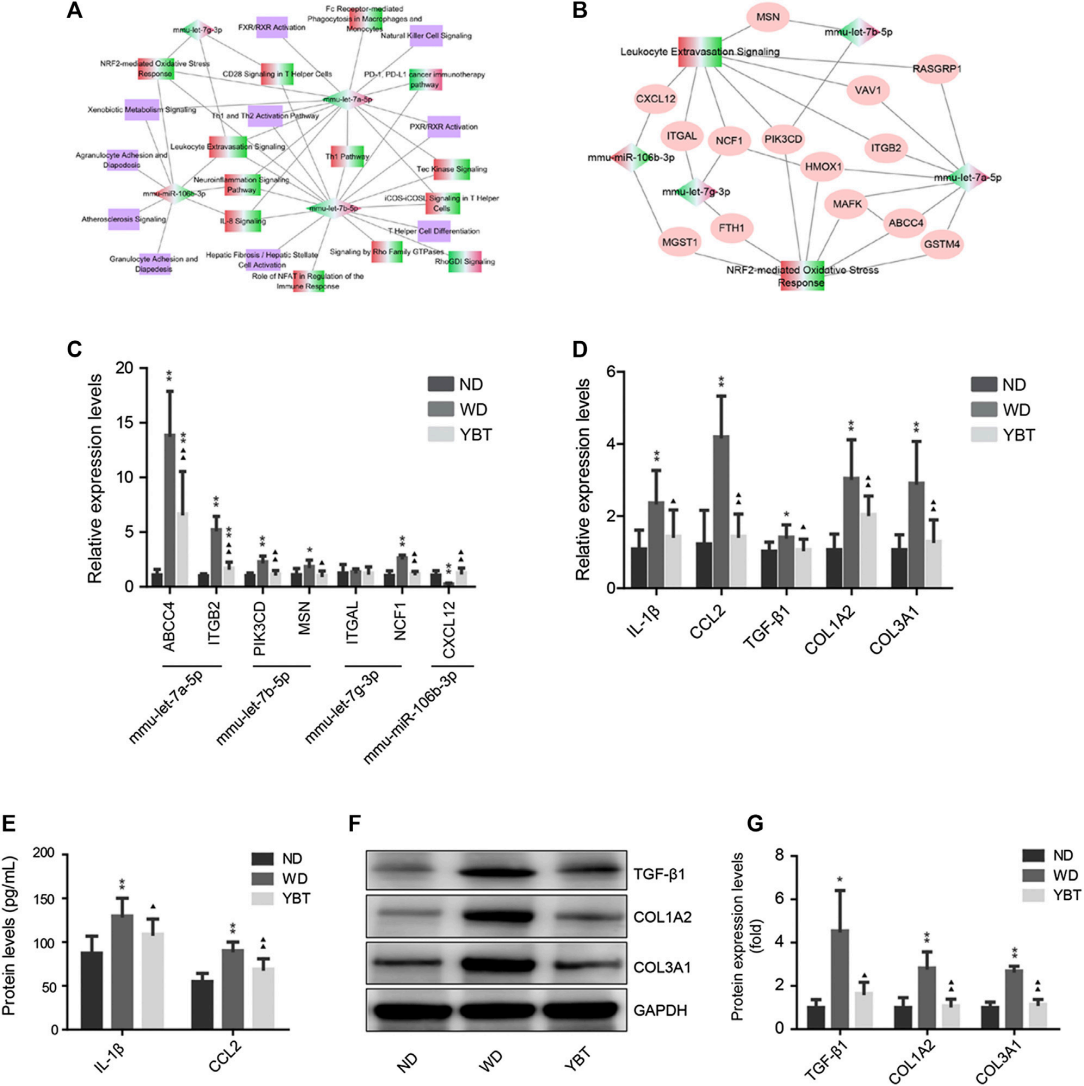
qRT-PCR verification of potential target genes, inflammation and fibrosis related genes. **(A)**. Simplified target MicroRNAs (miRNA)-pathway network obtained from panel 7B. The diamond denotes the target miRNA and the rectangle represents the canonical pathway. The red-green color presents the node was up-regulated in Western diet (WD) vs. normal diet (ND) group and down-regulated in Yiqi-Bushen-Tiaozhi (YBT) vs. WD group; the green-red color indicates the opposite situation. The purple color denotes the z-score of the pathway was unknown. **(B)**. Target miRNA-pathway network of Leukotyte Excetravation Sinaling Pathway and NRF2-mediated Oxiedativ Stress Response Pathway. The diamond denotes the target miRNA and the rectangle represents the canonical pathway. The ovals represent the potential target gene of the miRNAs. The red-green color presents the node was up-regulated in WD vs. ND group and down-regulated in YBT vs. WD group; the green-red color indicates the opposite situation. **(C)**. qRT-PCR verification of partial potential target genes of panel 8B. β-actin was used as the internal control. **(D)**. qRT-PCR verification of inflammation and fibrosis related genes. β-actin was used as the internal control. **(E)**. The protein levels of IL-1β and CCL-2 were measured by liquid suspension array analysis. **(F,G)**. Western blot analysis of the protein expression levels of TGF-βx, COL1A2 and COL3A1. GAPDH was served as the loading control. Compared with ND group, ^∗^ *p* < 0.05, ^∗∗^ *p* < 0.01; compared with WD group, ^▲^ *p* < 0.05, ^▲▲^ *p* < 0.01; n = 8/group.

To better understand the potential roles of these four miRNAs during YBT against NASH, we simplified Figure 7B into the target miRNA-pathway network which consisted of the four target miRNAs and their regulatory canonical pathways (Figure 8A) then classified the pathways (Table 5). Table 5 illustrated that most of the pathways belonged to inflammation/immunity (15 pathways), followed by cellular functions and signal transduction (four pathways). The remaining were xenobiotic metabolism (one pathway), oxidative stress (one pathway), liver fibrosis (1 pathway) and atherosclerosis (one pathway) associated pathways. Next, we generated the corresponding relationship between the target miRNAs and the classifications to describe the potential functions of the target miRNAs (Table 6).

**TABLE 5:** Classification of the canonical pathways regulated by the target miRNAs

Target miRNA	Canonical pathway	Classification
mmu-let-7a-5p	Fc Receptor-mediated Phagocytosis in Macrophage and Monocytes	Inflammation/immunity
mmu-let-7a-5p	Natural Killer Cell Signaling	Inflammation/immunity
mmu-let-7a-5p	FXR/RXR Activation	Cellular functions and signal transduction
mmu-let-7a-5p,mmu-let-7b-5p	PD-1, PD-L1 cancer immunotherapy pathway	Inflammation/immunity
mmu-let-7a-5p,mmu-let-7b-5p	PXR/RXR Activation	Cellular functions and signal transduction
mmu-let-7a-5p,mmu-let-7b-5p	Tec Kinase Signaling	Inflammation/immunity
mmu-let-7a-5p,mmu-let-7b-5p	iCOS-iCOSL Signaling in T Helper Cells	Inflammation/immunity
mmu-let-7a-5p,mmu-let-7b-5p	Th1 Pathway	Inflammation/immunity
mmu-let-7a-5p,mmu-miR-106b-3p	IL-8 Signaling	Inflammation/immunity
mmu-let-7a-5p,mmu-let-7b-5p,mmu-miR-106b-3p	Neuroinflammation Signaling Pathway	Inflammation/immunity
mmu-let-7a-5p,mmu-let-7b-5p,mmu-let-7g-3p	—	—
mmu-miR-106b-3p	Leukocyte Extravasation Signaling	Inflammation/immunity
mmu-let-7a-5p,mmu-let-7b-5p	Th1 and Th2 Activation Pathway	Inflammation/immunity
mmu-let-7a-5p,mmu-miR-106b-3p	Xenobiotic Metabolism Signaling	Xenobiotic metabolism
mmu-let-7a-5p,mmu-let-7b-5p,	—	—
mmu-let-7g-3p,mmu-miR-106b-3p	NRF2-mediated Oxidative Stress Response	Oxidative Stress
mmu-let-7b-5p	T Helper Cell Differentiation	Inflammation/immunity
mmu-let-7b-5p	RhoGDI Signaling	Cellular functions and signal transduction
mmu-let-7b-5p	Signaling by Rho Family GTPases	Cellular functions and signal transduction
mmu-let-7b-5p	Role of NFAT in Regulation of the Immune Response	Inflammation/immunity
mmu-let-7b-5p	Hepatic Fibrosis/Hepatic Stellate Cell Activation	Liver fibrosis
mmu-let-7b-5p,mmu-let-7g-3p,	CD28 Signaling in T Helper Cells	Inflammation/immunity
mmu-miR-106b-3p	Atherosclerosis signaling	Atherosclerosis
mmu-miR-106b-3p	Agranulocyte Adhesion and Diapedesis	Inflammation/immunity
mmu-miR-106b-3p	Granulocyte Adhesion and Diapedesis	Inflammation/immunity

miRNA, MicroRNAs.

**TABLE 6:** Corresponding relationships between the target MicroRNAs and the pathway classifications.

Target miRNA	Pathway classification	Canonical pathway
mmu-let-7a-5p	Inflammation/immunity	10
	Cellular functions and signal transduction	2
	Oxidative Stress	1
	Xenobiotic metabolism	1
mmu-let-7b-5p	Inflammation/immunity	11
	Cellular functions and signal transduction	3
	Oxidative Stress	1
	Liver fibrosis	1
mmu-let-7g-3p	Inflammation/immunity	2
	Oxidative Stress	1
mmu-miR-106b-3p	Inflammation/immunity	5
	Xenobiotic metabolism	1
	Oxidative Stress	1
	Atherosclerosis	1

Intriguingly, the result revealed that the four target miRNAs are all related to inflammation/immunity and oxidative stress. To verify the accuracy of the prediction, the alterations in the expression tendency of the corresponding target genes need to be detected. We constructed miRNA-target gene-pathway network (Figure 8B) from the two pathways with the highest connectivity degrees in Figure 8A, Leukotyte Excetravation Sinaling and NRF2-mediated Oxidative Stress Response, then selected one or two potential miRNA target gens for qRT-PCR verification. As shown in Figure 8C, the expression of ABCC4, ITGB2, PIK3CD and MSN, the potential target genes of mmu-let-7a-5p and mmu-let-7b-5p, increased greatly in NASH mice and decreased significantly in YBT treated mice. Among the two potential target genes of mmu-let-7g-3p, although the expression level of ITGAL did not change significantly in each group, the expression level of NCF1 was significantly enhanced in NASH mice and decreased significantly in YBT treated mice. The expression of CXCL12, a potential target gene of mmu-miR-106b-3p, was significantly reduced in NASH mice and increased significantly in YBT treated mice. The result indicated that the expression trend of most of the potential target genes were opposite to that of the hub miRNAs showed in Figure 7C, suggesting that the prediction result has a relatively high accuracy.

ABCC4 participates in bile acid transport, which is related to oxidative stress, inflammation (Allen et al., 2011) and fibrosis (Suga et al., 2019). It was found that the expression of ABCC4 was increased significantly during the development of NASH (Suga et al., 2019). ITGB2 participates in cell adhesion, which plays an important role in the inflammatory response in NAFLD (Hou et al., 2018). Interestingly, our result showed that the expression of ABCC4 and ITGB2 were strongly increased in NASH mice (relative expression levels 13.86 ± 4.02 and 5.22 ± 1.22, respectively) and was significantly decreased in YBT treated mice (relative expression levels 6.68 ± 3.86 and 1.70 ± 0.56, respectively). The expression trends of ABCC4 and ITGB2 were opposite to those of mmu-let-7a-5p, suggesting YBT may alleviate the abnormal expression of ABCC4 and ITGB2 via mmu-let-7a-5p.

PIK3CD encodes the catalytic subunit p110δ of phosphoinositide 3-kinase (PI3K). Previous studies on p110δ mutant mice showed that p110δ-Akt pathway plays a key role in HFD-induced inflammatory response (Mauro et al., 2017). High PI3Kδ expression level was found high in HCC and it plays significant roles in HCC (Ko et al., 2018). MSN encodes the moesin protein. Early studies showed that MSN deficient mice displayed significant reduced inflammatory infiltrate and fibrosis in liver (Okayama et al., 2008). Our research showed that PIK3CD and MSN expression levels are significantly increased in NASH mice and declined after YBT treatment. The expression trends of PIK3CD and MSN were opposite to those of mmu-let-7b-5p, suggesting that YBT may ameliorate diet-induced abnormal expression of PIK3CD and MSN via mmu-let-7b-5p.

NCF1 encodes the regulatory subunits of the NADPH oxidase complex, p47phox (Kong et al., 2019). NCF1- derived ROS play an important role in liver fibrosis and loss of NCF1 function in mice results in resistant to liver fibrosis (Bataller et al., 2003). In our study, the expression of NCF1 was up-regulated in NASH mice and was down-regulated after YBT intervention. The expression trend of NCF1 was opposite to that of mmu-let-7g-3p, suggesting that YBT may down-regulate NCF1 via mmu-let-7g-3p.

CXCL12 is the ligand of CXCR4 and CXCR7, The CXCL12/CXCR4 pathway contributes to the enhanced recruitment of CD4^+^ T-cells in NASH (Boujedidi et al., 2015). In the present study, the expression level of CXCL12 in NASH mice was strongly decreased and was significantly increased in YBT treated mice. The expression trend of CXCL12 was opposite to that of mmu-miR106-3p. The result revealed that YBT may participate in the regulation of CXCL12 through mmu-miR-106b-3p.

The above results showed that the hub miRNAs and their potential target genes are involved in immunity/inflammation or oxidative response. It is beyond doubt that inflammation and oxidative stress are vital mechanisms in the formation and progression of NASH (Li et al., 2016). Inflammation is one of the hallmarks of NASH and innate immune activation is a core factor in triggering and amplifying liver inflammation in NAFLD/NASH (Arrese et al., 2016). Oxidative stress plays a key role in the occurrence of inflammation (Masarone et al., 2018). Therefore, intervention on inflammation/immunity and oxidative stress are the two main directions of NASH prevention or treatment. We have found that, YBT recipe restrains hepatic oxidative stress induced by WD (Yang et al., 2019). In order to detect whether YBT contributed to suppression of inflammation, the inflammatory factors and fibrosis related genes were analyzed by qRT-PCR. As shown in Figure 8D, the mRNAexpression levels of the inflammatory factors (IL-1β1 and CCL-2) and fibrosis related genes (TGF-β1,COL1A2, and COL3A1) were significantly increased in NASH mice and significantly decreased in YBT treated mice. The liquid suspension array or Western blot analysis showed that the protein expression levels were consistent with that of mRNA expression levels (Figures 8E–G).The results revealed that YBT recipe attenuated inflammation in NASH mice.

**FIGURE 9:**
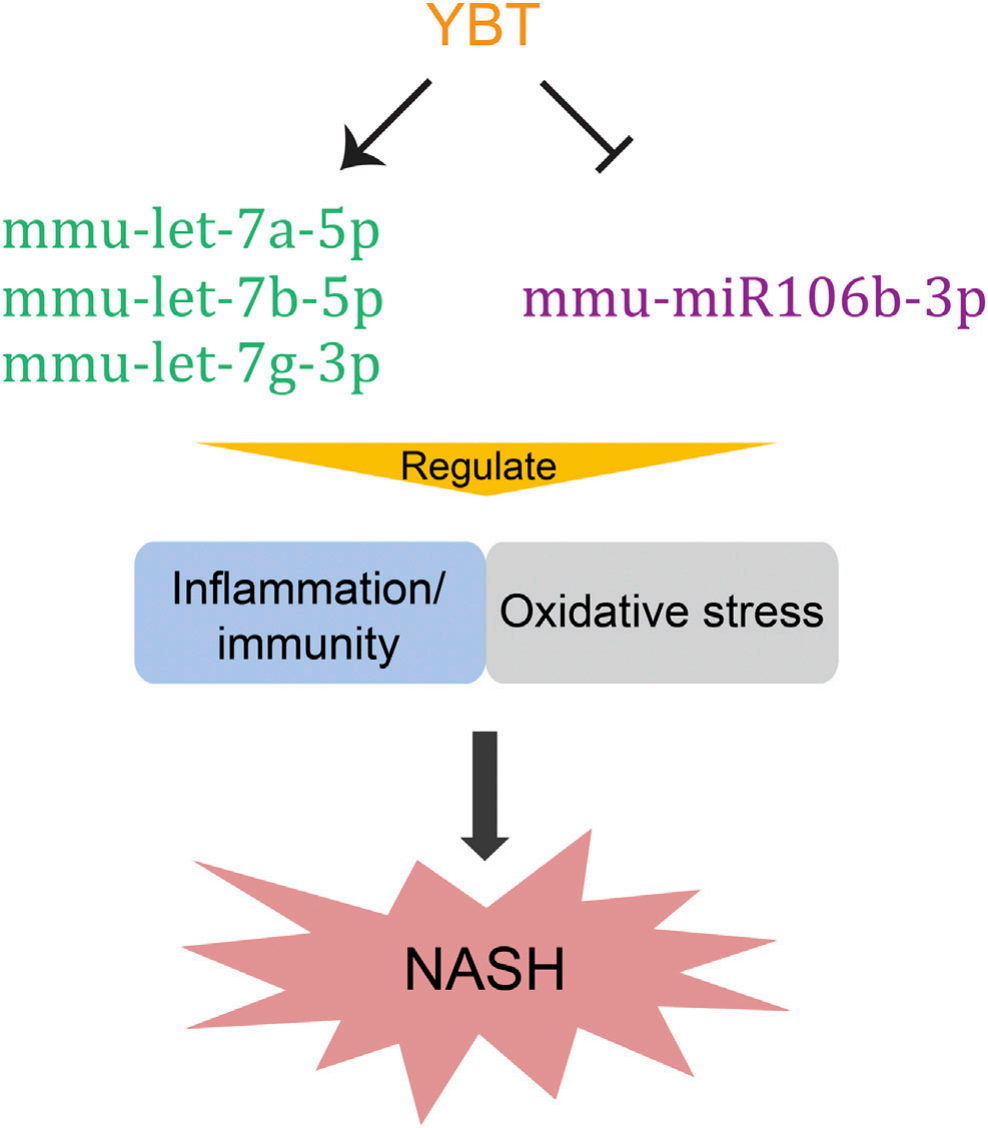
Potential underlying mechanisms of Yiqi-Bushen-Tiaozhi against non-alcoholic steatohepatitis. The arrow donates promotion; the T-bar represents inhibition.

A growing body of evidence suggests that flavonoids has a good effect on inhibiting chronic inflammation and oxidation (Perez-Cano and Castell, 2016). Isoflavonoids are a class of flavonoid phenolic compounds. So they have similar properties of anti-inflammation and antioxidation (Jheng et al., 2020). Our UHPLC-MS/MS analysis showed that there were 12.9 and 7.6% were classifiec as flavonoids and isoflavonoids, respectively. Therefore, our research suggested that, YBT might alleviate inflammation/immunity and oxidative stress in WD induced NASH by regulating mmu-let-7a-5p, mmu-let-7b-5p, mmu-let-7g-3p and mmu-miR106b-3p (Figure 9), which may be contributed by the components of flavonoids and isoflavonoids. Certainly, these findings need to be verified by further biological investigations. Although network pharmacology has its limitations and cannot replace biological function validation, this study provides useful information for guiding future studies on the mechanism of YBT against NASH by regulating miRNAs.

## Data Availability Statement

The miRNA raw data were uploaded to the Gene Expression Omnibus (GEO) database (https://www.ncbi.nlm.nih.gov/geo/query/acc.cgi?acc=GSE144721).

## Ethics Statement

The animal study was reviewed and approved by the Ethics Committee of Zhejiang Chinese Medical University (Resolution number ZSLL-2016-139).

## Author Contributions

WH and ZC designed the project. WH analyzed the data and wrote the manuscript. SL performed qRT-PCR and analyzed the data. YC performed qRT-PCR. TZ, QY and JY performed the animal experiments. TZ performed liquid suspension array analysis and Western blot assay. BH performed the biochemistry experiments. ZC supervised the project and revised the manuscript. All authors read and approved the final manuscript.

## Funding

Zhejiang Provincial Natural Science Foundation (LY15H270010); Zhejiang Traditional Chinese Medicine Science and Technology Plan Project (2016ZA087); Zhejiang Provincial Natural Science Foundation (LY17H290007); National Natural Science Foundation of China (81973610), National Natural Science Foundation of China (81673706).

## Conflict of Interest

The authors declare that the research was conducted in the absence of any commercial or financial relationships that could be construed as a potential conflict of interest.
