# Supplementary material for: The Target MicroRNAs and Potential Underlying Mechanisms of Yiqi-Bushen-Tiaozhi Recipe against‐Non-Alcoholic Steatohepatitis
Source: Front Pharmacol. 2020 Nov 12;11:529553. doi: 10.3389/fphar.2020.529553 (PMC7688626; doi:10.3389/fphar.2020.529553)
Supplement: Supplementary file 1 [file datasheet1.docx]

Supplementary Materials

# Supplementary Figures

#
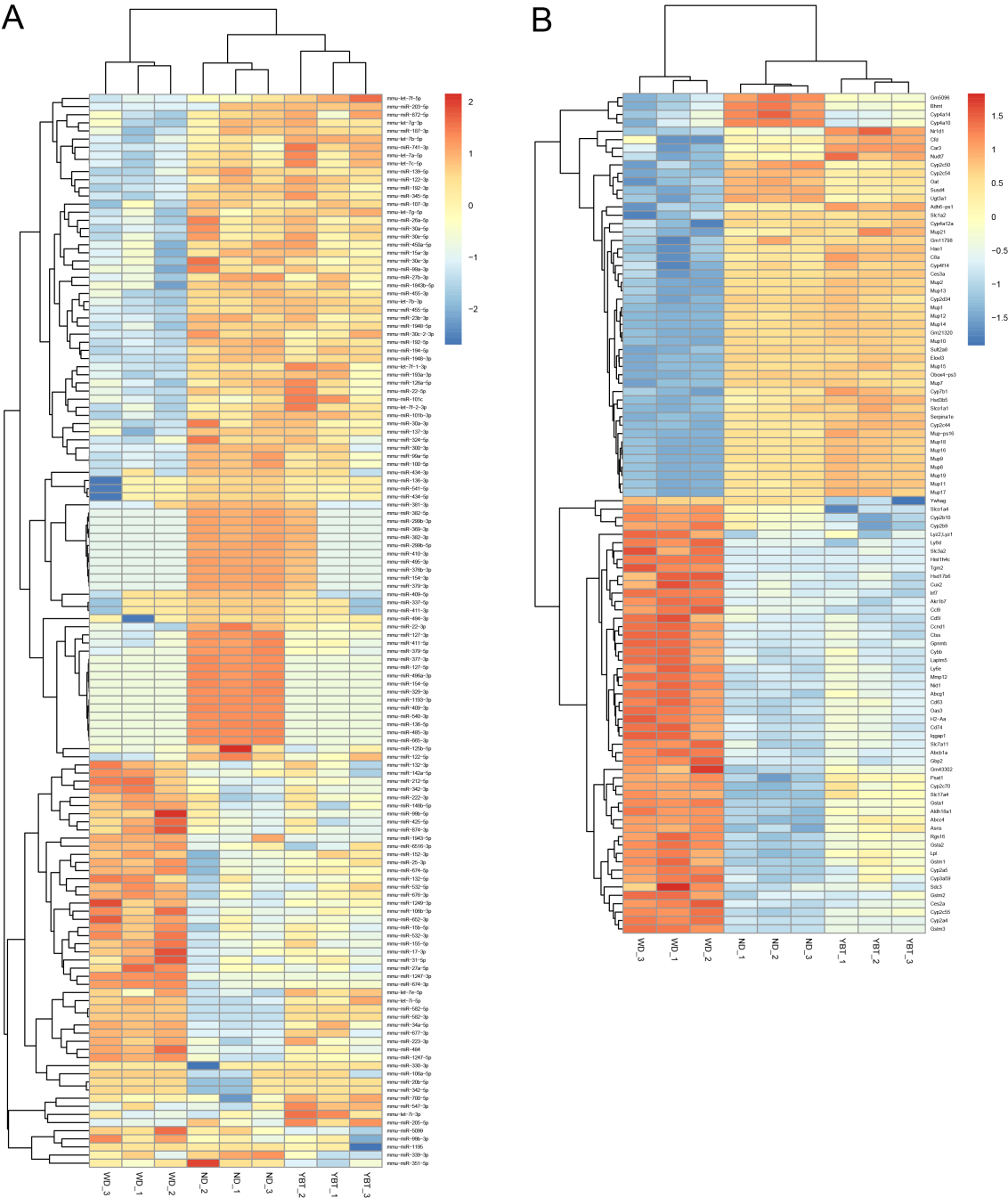


**Supplementary Figure 1.** Clustering heatmap of DEmiRNAs and DEmRNAs between ND, WD and YBT groups. (A). Heatmap of DEmiRNAs (*P* <0.05). (B). Heatmap of top 100 DEmRNAs (*P* <0.05). Scale bar denotes Z values. DEmiRNAs, significantly differentially expressed miRNAs; DEmRNAs, significantly differentially expressed mRNAs; ND, normal diet for 16-week; WD, Western diet for 16-week; YBT, Western diet for 16 weeks combined with YBT treatment for 12 weeks.


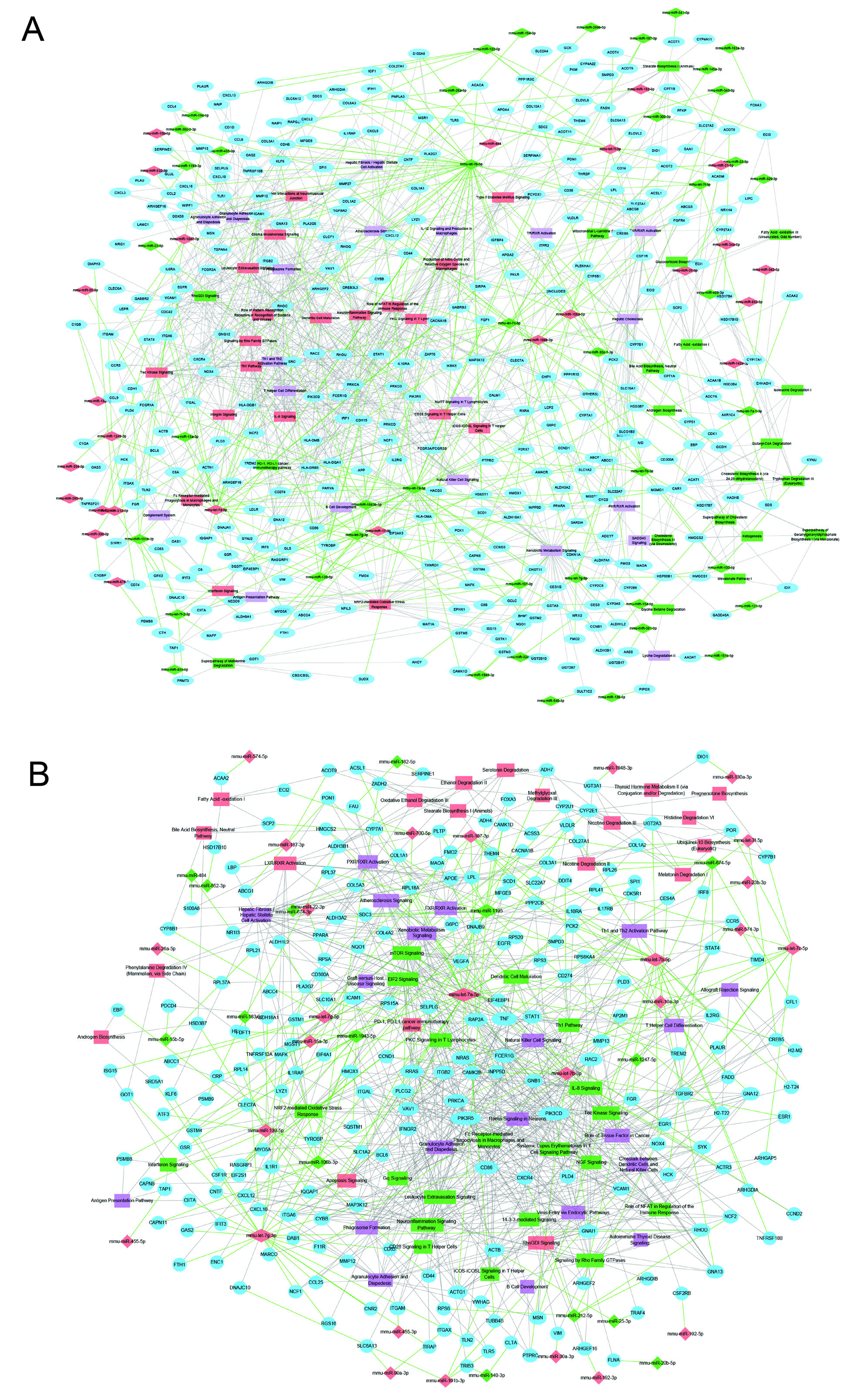


**Supplementary Figure 2.** miRNA-target gene-pathway networks of WD vs. ND group  and YBT vs. WD group. (A).The network of WD vs. ND group. (B). The network of YBT vs. WD group. The diamond, round or rectangle node indicates DEmiRNAs, target gene or canonical pathway, respectively. Red indicates up-regulation or z-score﹥0; blue indicates down-regulation or z-score﹤0; purple indicates z-score unknown. The green edge indicates the connection of DEmiRNA and its target gene; the grey edge denotes the connection between the target gene and the canonical pathway.

# Supplementary tables

**Table S1.** Result of UHPLC-MS/MS analysis.

**Table S2**. Significantly differentially expressed miRNAs in WD vs. ND group and YBT vs. WD group.

**Table S3**. Significantly differentially expressed mRNAs in WD vs. ND group and YBT vs. WD group.

**Table S4**. Information of the overlapping DEmRNAs.

**Table S5.** Information of the canonical pathways enriched by IPA.

**Table S6**. Top 60 canonical pathways.

**Table S7**. The 33 common canonical pathways between WD vs. ND group and YBT vs. WD group.

**Table S8**. Nodes information of the miRNA-target gene-pathway networks.

**Table S9**. Count-scores of the DEmiRNA-canonical pathway pairs.

**Table S10**. Note information of the miRNA-pathway networks.

**Table S11**. Primers of stem-loop qRT-PCR.
