# Supplementary material for: The Target MicroRNAs and Potential Underlying Mechanisms of Yiqi-Bushen-Tiaozhi Recipe against‐Non-Alcoholic Steatohepatitis
Source: Front Pharmacol. 2020 Nov 12;11:529553. doi: 10.3389/fphar.2020.529553 (PMC7688626; doi:10.3389/fphar.2020.529553)
Supplement: Supplementary file 2 [file datasheet2.zip › Data sheets 0930/Table S7.docx]

**Table S7. The 33 common canonical pathways between WD vs. ND group and YBT vs. WD group.**

|  | **WD vs. ND** | | | **YBT vs. WD** | | |
| --- | --- | --- | --- | --- | --- | --- |
| **Canonical pathway** | **-log(p-value)** | **Ratio** | **z-score** | **-log(p-value)** | **Ratio** | **z-score** |
| Agranulocyte Adhesion and Diapedesis | 3.09 | 0.139 | -- | 4.43 | 0.133 | -- |
| Androgen Biosynthesis | 2.94 | 0.385 | -2.236 | 2.48 | 0.308 | 2 |
| Antigen Presentation Pathway | 4.21 | 0.321 | -- | 8.39 | 0.429 | -- |
| Atherosclerosis Signaling | 5.94 | 0.207 | -- | 6.13 | 0.18 | -- |
| B Cell Development | 2.67 | 0.25 | -- | 4.26 | 0.286 | -- |
| Bile Acid Biosynthesis, Neutral Pathway | 5.43 | 0.583 | -2.646 | 2.62 | 0.333 | 2 |
| CD28 Signaling in T Helper Cells | 2.82 | 0.15 | 2.111 | 3.22 | 0.133 | -2.828 |
| Dendritic Cell Maturation | 5.04 | 0.169 | 4.2 | 8.03 | 0.175 | -4.6 |
| Fatty Acid β-oxidation I | 10.4 | 0.5 | -4 | 3.02 | 0.219 | 2.646 |
| Fcγ Receptor-mediated Phagocytosis in Macrophages and Monocytes | 6.2 | 0.228 | 3.71 | 6.82 | 0.207 | -3.9 |
| FXR/RXR Activation | 8.06 | 0.235 | -- | 4.15 | 0.148 | -- |
| Granulocyte Adhesion and Diapedesis | 5.31 | 0.174 | -- | 5.97 | 0.155 | -- |
| Hepatic Fibrosis / Hepatic Stellate Cell Activation | 3 | 0.134 | -- | 4.38 | 0.128 | -- |
| iCOS-iCOSL Signaling in T Helper Cells | 4.67 | 0.19 | 2.324 | 4.1 | 0.152 | -3.051 |
| IL-8 Signaling | 5.78 | 0.168 | 2.785 | 7.44 | 0.157 | -2.785 |
| Interferon Signaling | 2.4 | 0.226 | 2.646 | 3.11 | 0.226 | -2.646 |
| Leukocyte Extravasation Signaling | 5.23 | 0.161 | 3.9 | 6.26 | 0.145 | -4.315 |
| Natural Killer Cell Signaling | 2.84 | 0.155 | -- | 4.2 | 0.155 | -- |
| Neuroinflammation Signaling Pathway | 6.25 | 0.153 | 3.43 | 7.18 | 0.135 | -3.536 |
| NRF2-mediated Oxidative Stress Response | 5.83 | 0.171 | 3.153 | 5.26 | 0.138 | -2.714 |
| PD-1, PD-L1 cancer immunotherapy pathway | 1.61 | 0.128 | -2.309 | 4.71 | 0.17 | 2.5 |
| PKCθ Signaling in T Lymphocytes | 1.63 | 0.114 | 3 | 3.68 | 0.128 | -3.771 |
| PXR/RXR Activation | 7.33 | 0.3 | -- | 5.86 | 0.233 | -- |
| RhoGDI Signaling | 2.18 | 0.121 | -2 | 3.28 | 0.116 | 1.604 |
| Role of NFAT in Regulation of the Immune Response | 2.21 | 0.122 | 2.524 | 4.66 | 0.134 | -2.985 |
| Signaling by Rho Family GTPases | 2.02 | 0.11 | 2.837 | 2.33 | 0.0932 | -3.357 |
| Stearate Biosynthesis I (Animals) | 6.89 | 0.333 | -2.138 | 2.14 | 0.156 | 1.134 |
| T Helper Cell Differentiation | 3.89 | 0.209 | -- | 6.76 | 0.239 | -- |
| Tec Kinase Signaling | 3.44 | 0.146 | 3.3 | 4.81 | 0.14 | -3.873 |
| Th1 and Th2 Activation Pathway | 3.89 | 0.154 | -- | 5.92 | 0.154 | -- |
| Th1 Pathway | 3.87 | 0.173 | 2.324 | 4.97 | 0.164 | -3.742 |
| Xenobiotic Metabolism Signaling | 7.64 | 0.167 | -- | 5.31 | 0.121 | -- |

Ratio, the proportion of the DEmRNAs in the total genes of the pathways. Short horizontal line, the z-score was unkown. *P*﹤0.05.
